# Fasudil Alleviates Postoperative Neurocognitive Disorders in Mice by Downregulating the Surface Expression of α5GABAAR in Hippocampus

**DOI:** 10.1111/cns.70098

**Published:** 2024-11-03

**Authors:** Jinpeng Dong, Zhun Wang, Lixuan Li, Mengxue Zhang, Sixuan Wang, Yuan Luo, Ying Dong, Xiaokun Wang, Yongan Wang, Kaiyuan Wang, Yiqing Yin

**Affiliations:** ^1^ Department of Anesthesiology, Key Laboratory of Cancer Prevention and Therapy, State Key Laboratory of Druggability Evaluation and Systematic Translational Medicine, National Clinical Research Center for Cancer Tianjin Medical University Cancer Institute and Hospital, Tianjin's Clinical Research Center for Cancer Tianjin China; ^2^ Jinan University Guangzhou China; ^3^ State Key Laboratory of Toxicology and Medical Countermeasures, Academy of Military Medical Sciences Beijing China

**Keywords:** calcium‐dependent protein kinase II, fasudil, postoperative neurocognitive disorders, radixin, α5GABAA receptors

## Abstract

**Aim:**

Postoperative neurocognitive disorder (PND) refers to the cognitive impairment experienced by patients after surgery. As a target of sevoflurane, a kind of inhalation anesthetic, the balance of the GABAergic system can be disrupted during the perioperative period. In this study, we explored the promoting effect of abnormal elevation of the α5 subtype of γ‐aminobutyric acid type A (GABAA) receptors caused by sevoflurane and surgical trauma on PND, as well as the therapeutic effect of fasudil on PND.

**Methods:**

Eight‐week‐old mice were pretreated with fasudil, and after 10 days, sevoflurane‐induced femoral fracture surgery was performed to establish an animal model of PND. The Morris water maze and fear conditioning tests were used to evaluate PND induced by this model. Biochemical and electrophysiological analyses were conducted to assess the protective effect of fasudil on the GABAergic system.

**Results:**

Following artificial fracture, the hippocampus‐dependent memory was damaged in these mice. Fasudil pretreatment, however, ameliorated cognitive function impairment in mice induced by sevoflurane and surgery. Mechanistically, fasudil was found to restore the increased hippocampus expression and function of α5GABAARs in mice with PND. In addition, pretreatment with Fasudil inhibited the enhancement in the calcium ion concentration and phosphorylation of Camk2, as well as the activation of the Radixin pathway which led to increased phosphorylation of the ERM family in the hippocampal CA1 region of the PND model.

**Conclusion:**

Preadministration of fasudil improved postoperative cognitive function in PND mice by inhibiting the activation of Camk2 and Radixin pathways and finally downregulating the surface expression of α5GABAAR in hippocampus neurons.

## Graphical abstract

Anesthesia and surgery increase the calcium concentration in the hippocampus, increasing the phosphorylation of Camk2. Anesthesia and surgery activate the Radixin pathway, leading to increased phosphorylation of Radixin. The activation of these two pathways results in increased surface expression of α5GABAARs, ultimately leading to hippocampus‐dependent memory impairment. Preadministration of the neuroprotective drug fasudil can inhibit the phosphorylation of the key molecules mentioned above, prevent the translocation of α5GABAARs to the cell surface, and thereby mitigate neurocognitive damage.

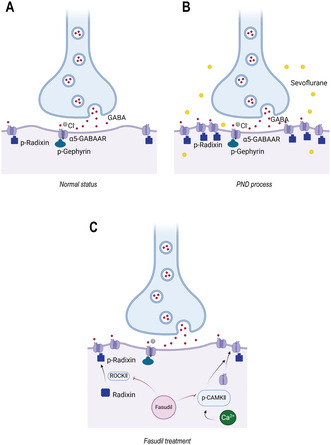

## 1 Introduction

Postoperative neurocognitive disorder (PND) is a term proposed by anesthesia and neurology experts worldwide to replace the former term postoperative cognitive dysfunction (POCD) [1]. PND is one of the most common postoperative complications in patients and is characterized by impaired memory and attention during the postoperative period [1, 2]. In a prospective study, the incidence of postoperative neurocognitive dysfunction at discharge was 34.5% among 1064 adult noncardiac surgical patients [3]. The resulting decline in cognitive function can persist for several months or years and can have adverse effects on independence, quality of life, the risk of developing dementia, and even long‐term survival. This high incidence of central nervous system complications not only affects patients’ recovery time and quality of life but also adds additional economic burden on families and society [4]. Therefore, seeking interventions for PND is necessary.

Postoperative neurocognitive disorder is a complex condition influenced by various factors, with a significant focus on the role of anesthetics and neuroinflammation in its development [2, 5, 6, 7, 8, 9, 10]. The γ‐aminobutyric acid (GABA) receptors, particularly the type A receptor (GABAAR), have attracted considerable attention due to their involvement in PND pathogenesis [11, 12, 13, 14, 15]. GABAAR is a key inhibitory neurotransmitter receptor in the brain, with distinct distributions and functions across different subtypes [16]. The α5GABAAR subtype, primarily located in the hippocampal region and predominantly found at extrasynaptic sites on neuronal membranes, plays a crucial role in PND [17]. When GABA binds to α5GABAAR, it triggers the opening of Cl^−^ channels, leading to membrane hyperpolarization known as tonic inhibition, reflecting the functionality of postsynaptic α5GABAAR [18]. Research has highlighted the aberrant upregulation of α5GABAAR function in the hippocampus as a pivotal factor in PND development [10, 19, 20, 21, 22, 23, 24].

The anchoring of α5GABAAR to the cell membrane involves intracellular scaffold molecules, with Radixin serving as the specialized scaffold for extrasynaptic α5GABAAR. Radixin, a member of the Ezrin/Radixin/Moesin (ERM) family of cytoskeletal proteins, interacts with transmembrane proteins and requires phosphorylation, dependent on Rho GTPase signaling, for its interaction with α5GABAAR [25, 26]. Studies have demonstrated that increased Radixin phosphorylation leads to the clustering of α5GABAARs at extrasynaptic sites, contributing to cognitive impairment in mice [27]. Additionally, sevoflurane administration has been shown to induce excessive extrasynaptic clustering of α5GABAAR by enhancing Radixin phosphorylation in aged mice [28]. Inhalational anesthetics can activate the ryanodine receptor, causing an increase in intracellular calcium ion concentration, which, in turn, triggers the Ca^2+^/Camk2 pathway [29, 30, 31]. This pathway plays essential roles in synaptic transmission, learning, memory, and cellular functions in neurons by promoting the insertion of GABAAR subunits into the cell membrane through Camk2 phosphorylation [32, 33].

Fasudil, which was approved as a neuroprotective agent in Japan and China in 1995 [34], exerts dual pharmacological inhibitory effects on ROCK and Camk2 [35]. The inhibitory effect of fasudil on ROCK has been utilized in the treatment of cerebral vasospasm due to subarachnoid hemorrhage and has shown potential for improving cognitive decline in stroke patients [36, 37]. Studies have indicated that fasudil can enhance memory in mice, with this improvement occurring under physiological conditions, as well as in the protection of cognitive function following Alzheimer's disease and epilepsy [38, 39, 40]. However, its effects on PND remain unknown. It is hypothesized that fasudil inhibits the ROCK II/Radixin pathway activated by sevoflurane and surgical trauma, preventing Radixin phosphorylation and the accumulation of α5GABAARs on the cell membrane, thus ameliorating cognitive deficits in postoperative mice. Thus, the current research aims to explore the potential role and mechanisms of fasudil in mitigating postoperative cognitive impairments using a mouse model of PND.

## 2 Materials and Methods

### 2.1 Animals

#### 2.1.1 Animal Care

Eight‐week‐old male C57BL/6 mice were purchased from SPF Biotechnology Co., Ltd. (Beijing, China). All the mice were housed in an SPF‐grade animal facility with an ambient temperature of 22°C ± 1°C and a humidity of 50%–60%. The light–dark cycle was maintained at 10/14 h. No more than five mice were housed in each cage, and both water and food were provided ad libitum, with bedding changed regularly. All experimental procedures were conducted according to the “Guide for the Care and Use of Laboratory Animals” published by the National Institutes of Health (NIH Publications No. 8023, revised in 1996) and the “ARRIVE guidelines.” The entire animal study was approved by the Ethics Committee for Animal Research of Tianjin Medical University Cancer Institute and Hospital, with ethical approval number WTW‐AE‐2024013.

#### 2.1.2 Animal Grouping

A total of 60 mice were randomly divided into the following four groups: the solvent + control group (NS + CON), fasudil + control group (FSD + CON), solvent + surgery group (NS + SUR), and fasudil + surgery group (FSD + SUR). Immediately after the completion of the surgical or control treatments, four mice were randomly selected from each group for blood collection from the abdominal aorta, followed by a blood gas analysis. The remaining mice in each group were randomly selected for the subsequent Morris water maze (MWM), fear conditioning test (FCT), and open field test. After all tests were completed, the mice were euthanized, and brains were removed, frozen and sectioned to collect the hippocampus. The remaining mice were kept for subsequent acute brain slice electrophysiology testing.

#### 2.1.3 Anesthesia and Surgery

We established an animal model via closed reduction internal fixation. The surgical group of mice was anesthetized with 4.3% sevoflurane (Shanghai Hengrui Pharmaceutical Co., Ltd., Shanghai, China) in a transparent acrylic chamber with 50% O_2_ + 50% air for 20 min, followed by placing the mice head in a mask through which 4.3% sevoflurane was administered at a gas flow rate of 1 L·min^−1^. The gas concentration monitoring equipment was customized by us at Zhongshi Technology (Zhongshi Science Technology, Beijing, China). Under sterile surgical conditions, a 0.45 mm diameter steel needle was inserted into the right femoral marrow cavity of each mouse, after which a midshaft‐closed femoral fracture was created via a self‐designed fracture model support, which was completed in a total of 10 min. Postoperatively, the mice were anesthetized in the above‐mentioned acrylic chamber for 90 min, and after anesthesia, lidocaine cream was applied for pain relief. The mice were subsequently allowed to recover for 30 min in a 50% O_2_ + 50% air environment before being placed back in their original cages. The nonsurgical (control) mice inhaled only 50% O_2_ + 50% air for 2 h in a transparent acrylic chamber at 37°C. Throughout the surgical procedure, we maintained the temperature of the chamber bottom at 37°C using a warming blanket and continuously monitored the vital signs, such as heart rate, blood oxygen saturation, and respiratory rate, of the mice during anesthesia or exposure to control gases. We also continuously monitored the concentrations of sevoflurane and carbon dioxide inside the acrylic chamber during this process. All procedures were conducted to ensure the comfort of the experimental animals during the surgical procedures. Sterile techniques were strictly followed during the surgery, and postoperatively, we applied bandages and local antibiotics to the operated mice (and control mice) on the affected side. These measures were taken to some extent to prevent infection.

#### 2.1.4 Drug Administration to Animals

Based on previous research [36, 37, 38], we determined that the duration of fasudil treatment should be 10 days, with a dosage of 15 mg·kg^−1^. The fasudil + control group (FSD + CON) and the fasudil + surgery group (FSD + SUR) received daily intraperitoneal injections of fasudil (Selleck, TX, USA). Fasudil was dissolved directly in saline solution before injection. The solvent + control group (NS + CON) and the solvent + surgery group (NS + SUR) received injections of saline solution. The timing of drug administration was consistent each day.

### 2.2 Primary Neuron Cultures and Treatment

**FIGURE 1:**
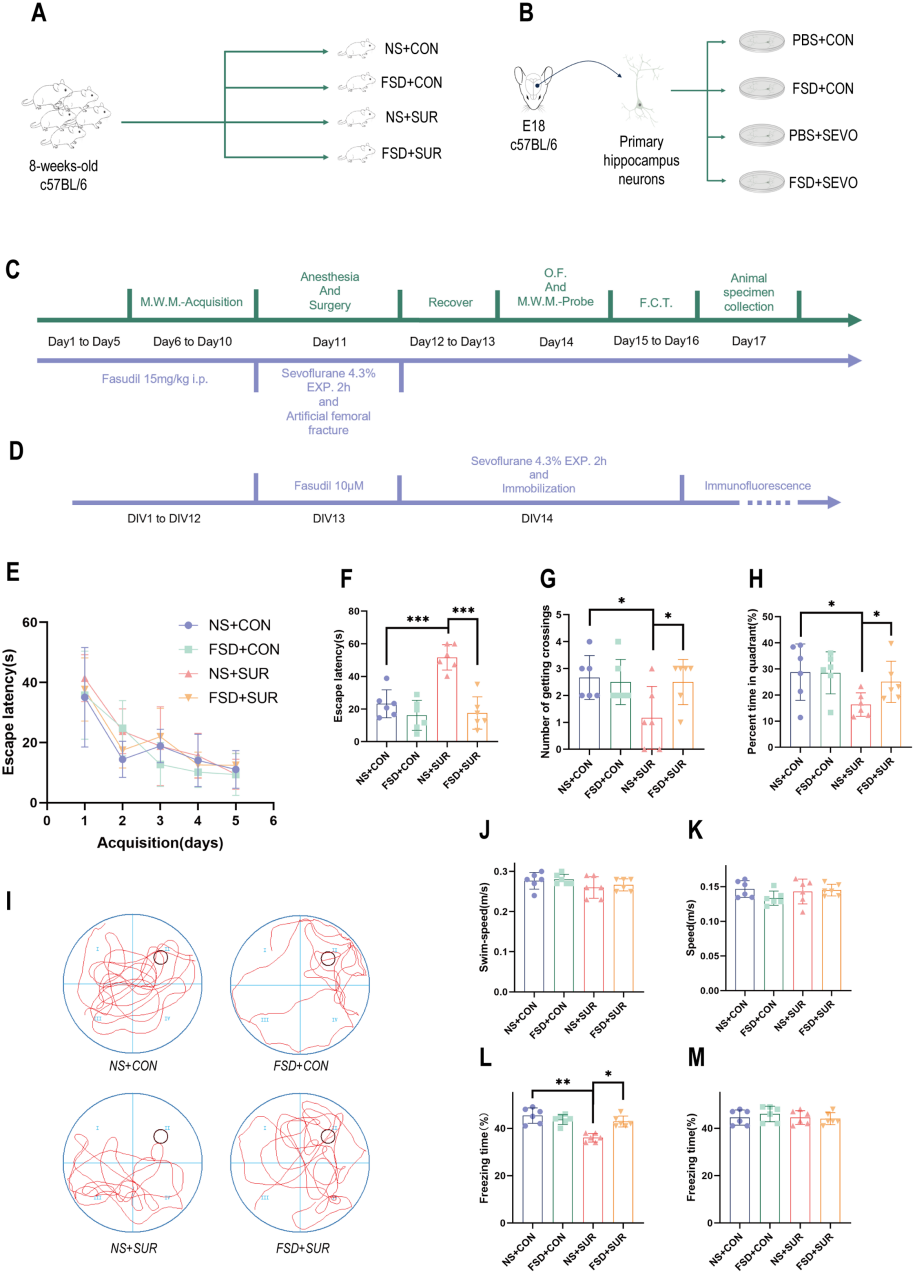
The establishment of the PND model and the results of the behavioral tests. (A–D) Establishment of mouse and primary neuronal models. (A) Mouse age, strain, and grouping. (B) Method for primary neuronal extraction and grouping. (C) Timeline for establishing and testing the mouse model. (D) Timeline for establishing and testing the primary neuronal model. (E) Line graph showing the latency period of the mice in the learning phase of the water maze test (*n* = 6). (F) Time taken by the mice to find the platform location during the probe test phase (*n* = 6). (G) Number of times the mice crossed the original platform location during the probe test phase (*n* = 6). (H) Time spent by the mice in the platform quadrant during the probe test phase (*n* = 6). (I) Swimming paths of the mice during the probe test phase. (J) Swimming speed of the mice during the probe test phase (*n* = 6). (K) Locomotor speed of the mice in the open field test (*n* = 6). (L) Contextual freezing time of the mice in the FCT (*n* = 6). (M) Freezing time of the mice in response to the conditioned tone in the FCT (*n* = 6). The data are presented as the means ± SEMs, *p < 0.05, **p < 0.01, and ***p < 0.001 (unpaired Student's *t*‐test).

Primary neurons were obtained from the hippocampi of C57BL/6 (E18) mice. The cell culture dishes were coated with 0.1 mg/mL poly‐L‐lysine and incubated overnight at 37°C. On the following day, the hippocampi of neonatal mice were dissected, minced, and transferred to 15 mL centrifuge tubes. Then, 5 mL of 0.2% collagenase and 5 mL of 0.25% trypsin were added, along with 5 μL of DNase, and the mixture was digested at 37°C for 15 min. After digestion, 20 mL of high‐glucose DMEM (GIBCO BRL, NY, USA) containing 10% fetal bovine serum (BioInd, Kibbutz Beit, Israel) was added to terminate the digestion. The solution was then passed through a 40 μm mesh filter and centrifuged, and the resulting cell pellet was resuspended in PBS for cell counting. After another centrifugation step, the cells were resuspended in high‐glucose DMEM and adjusted to a density of 6.5–7.0 × 10^5^ cells/ml. After 14 days of in vitro culture, the neurons were exposed to cell culture gas (95% air + 5% CO_2_) containing 4.3% sevoflurane for 2 h. The control group was exposed to cell culture gas (95% air + 5% CO_2_). In the intervention study, fasudil at a concentration of 10 μM in PBS was added to the cell culture medium 24 h before anesthesia exposure. The concentration of fasudil used was based on previous studies. All the groups were exposed for 2 h (Figure 1D).

### 2.3 HT‐22 Cell Culture and Treatment

HT‐22 cells (Cyagen Biosciences, Jiangsu, China) were cultured in DMEM containing 10% fetal bovine serum and 1% penicillin/streptomycin at 37°C in a humidified incubator with 5% CO_2_. Small interfering RNAs (siRNAs) targeting Radixin and Camk2 (Radixin‐siRNA, Camk2‐siRNA; Tsingke Biotechnology, Beijing, China) were transfected into the cells using Lipofectamine 3000 reagent (Thermo Fisher Scientific, Waltham, MA, USA). The cells were incubated at 37°C in a humidified incubator with 5% CO_2_ for 48 h before anesthesia exposure. Nontransfected cells were used as controls. In the intervention study, fasudil at a concentration of 10 μM in PBS was added to the cell culture medium 24 h before anesthesia exposure. All the groups were exposed for 2 h.

### 2.4 Open Field Test

On the third day after surgery, an open field test was conducted to assess the recovery of motor function in the mice. The mice were placed in the center of a 50 × 50 × 40 cm acrylic board setup, and a camera was used to track the movement trajectories of the animals in the peripheral and central areas of the open field arena. The test duration was 5 min. The movement speed of the mice during this period was then recorded and analyzed using VisuTrack video analysis software (Xinruan Technology, Shanghai, China).

### 2.5 Fear Condition Test

On the fourth day postsurgery, the mice underwent training for contextual fear conditioning. The mice were placed in the conditioning chamber for a 3‐min habituation period. Following habituation, a single tone–foot shock pair was administered (tone: 30 s, 70 dB, 1 kHz; foot shock: 2 s, 0.5 mA, with a 30‐s interstimulus interval). This stimulus was followed by two additional pairs of foot shocks. After the final shock, the mice remained in the chamber for 1 min before being returned to their home cages.

Twenty‐four hours after training, the mice were placed back in the same testing chamber for the assessment of contextual fear memory. The mice were allowed to freely explore the chamber for 6 min to establish a baseline freezing threshold. The percentage of time spent freezing was automatically identified and recorded using VisuTrack video analysis software.

Two hours later, an auditory fear memory was assessed by placing the mice in a novel environment resembling the training chamber. The mice were allowed to explore for 3 min before being presented with the same auditory stimuli used during training (30 s, 70 dB, 1 kHz, with a 1‐min interstimulus interval). Two additional sets of auditory stimuli were administered with no foot shocks. Freezing behavior was recorded and analyzed. Freezing behavior was defined as the absence of any visible movement except for respiration.

### 2.6 Morris Water Maze

The mice were trained for 5 days prior to surgery and tested on the third day postsurgery. Specifically, the water maze equipment consisted of a circular water pool with a diameter of 122 cm. The pool water was made milky white by adding white food coloring, and the water temperature was maintained at 23°C ± 2°C. A platform measuring 10 cm^2^ was placed in the pool, and the platform was submerged 0.5 cm below the water surface. During the experimental preparation phase, the water pool was divided into four quadrants, and the order of placing the mice into the water and platform locations can be found in Table 1. The latency period was defined as the time it took for the mouse to find and stay on the platform for at least 3 s, and if the mouse failed to find the platform within 60 s, the experimenter gently guided the mouse to the platform and allowed it to remain there for 30 s to reinforce memory. During the testing phase, the platform was removed, and the mice were placed into the water from the opposite side of the platform and allowed to swim for 60 s before being removed. Data such as the time spent by the mouse in the original platform quadrant within 60 s, the average swimming speed of the mouse, and the number of times the mouse crossed the original platform location were recorded using VisuTrack video analysis software.

**TABLE 1:** Sequence of placing mice in the water maze and platform.

Acquisition				
Day	Trial 1	Trial 2	Trial 3	Trial 4
1	N	E	SE	NW
2	SE	N	NW	E
3	NW	SE	E	N
4	E	NW	N	SE
5	N	SE	E	NW
6 (Probe)	NE			

### 2.7 Western Blot

After the mouse hippocampus was rapidly dissected, it was frozen in liquid nitrogen and stored at −80°C until use. Both total protein and membrane protein were extracted from the mouse hippocampus, after which the protein concentrations were quantified. The protein samples were separated on 10% SDS‐PAGE gels and transferred onto polyvinylidene fluoride (PVDF) membranes (Merck KGaA, Darmstadt, Germany). The membranes were blocked with rapid blocking solution (Yamei Biotechnology, Shanghai, China) at room temperature for 15 min and then incubated with primary antibodies overnight at 4°C on a shaker. The next day, the membranes were incubated with horseradish peroxidase (HRP)‐conjugated secondary antibodies at room temperature for 1 h. The antibodies used in this study included anti‐α5GABAAR (1:1000; ab259880; Abcam, MA, USA), anti‐p‐ERM (1:500; ab76247; Abcam), anti‐ERM (1:1000; ab231642; Abcam), anti‐GAPDH (1:50000; A19056; ABclonal, Wuhan, China), anti‐pCamk2 (1:1000; AP1386; ABclonal), anti‐Camk2 (1:1000; A0198; ABclonal), and anti‐ROCKII (1:1000; 21,645‐1‐AP; Proteintech, Wuhan, China) antibodies. All the antibodies were of rabbit origin. The protein bands were visualized using chemiluminescence (Pierce ECL Western blotting substrate; Thermo Fisher Scientific, MA, USA) and measured with a computer image analysis system (ChemiDoc XRS+; Bio‐Rad Hercules, CA, USA).

### 2.8 Immunofluorescence Staining

#### 2.8.1 Brain Slices

The mice were sequentially perfused with saline and 4% paraformaldehyde through the heart to obtain the whole brain. The whole‐brain samples were then fixed with a tissue fixation solution (Servicebio, Wuhan, China) on a shaker at room temperature for 1 day, followed by the preparation of frozen brain sections. The sections were stored at −80°C for later use. Before immunostaining, the brain sections were thawed at room temperature for 30 min. Then, the sections were blocked with an immunostaining blocking solution (Beyotime, Shanghai, China) at room temperature for 40 min. One set of brain sections was incubated with a mouse anti‐α5GABAAR antibody (1:100; ab302962; Abcam) overnight at 4°C. The next day, the sections were permeabilized with 0.3% Triton X‐100 at 37°C for 15 min. After blocking for 40 min, the sections were incubated with rabbit anti‐p‐ERM antibody (1:100; ab76247; Abcam) overnight at 4°C. The following day, the sections were incubated with an Alexa Fluor 555‐conjugated goat anti‐rabbit secondary antibody (1:200; ab300672; Abcam) and an Alexa Fluor 647‐conjugated goat anti‐mouse secondary antibody (1:200; ab150115; Abcam) at room temperature for 2 h, followed by DAPI (ab104139; Abcam) staining. Another set of brain sections was incubated with a rabbit anti‐pCamk2 antibody (1:50; AP1386; ABclonal) overnight at 4°C. The next day, the sections were incubated with an Alexa Fluor 555‐conjugated goat anti‐rabbit secondary antibody (1:200; ab300672; Abcam) for 2 h and then subjected to DAPI staining. Images were acquired using a laser‐scanning confocal microscope (LSM 880 with Airyscan, Carl Zeiss AG, Oberkochen, Germany).

#### 2.8.2 Primary Neurons

After the coverslips were removed, the cells were permeabilized with 0.3% Triton X‐100 at 37°C for 15 min and then blocked with an immunostaining blocking solution at room temperature for 40 min. Then, cells on one set of coverslips were incubated with a mouse anti‐α5GABAAR antibody (1:100; ab302962; Abcam) and a rabbit anti‐p‐ERM antibody (1:100; ab76247; Abcam) overnight at 4°C. Cells on another set of coverslips were incubated with a rabbit anti‐pCamk2 antibody (1:50; AP1386; ABclonal) overnight at 4°C. The next day, the cells on the coverslips were incubated with an Alexa Fluor 555‐conjugated goat anti‐rabbit secondary antibody (1:200; ab300672; Abcam) and an Alexa Fluor 647‐conjugated goat anti‐mouse secondary antibody (1:200; ab150115; Abcam) at room temperature for 2 h, followed by DAPI staining. Images were acquired using a laser scanning confocal microscope.

### 2.9 Measurement of Calcium Ion Concentrations in the Hippocampus

The mice were rapidly decapitated, and the hippocampus was dissected and the mass was measured using a precision balance. The calcium content within the hippocampus of different groups of mice was determined using a calcium content colorimetric assay kit (Beyotime, Shanghai, China).

### 2.10 In Vitro Electrophysiology

Whole‐cell recordings were performed on pyramidal neurons in the CA1 region of acute hippocampal slices from mice. Coronal slices were prepared using a vibratome (VT1000S; Leica, Wetzlar, Germany) with a slicing speed of 0.025 mm/s, an amplitude of 0.8 mm, and a slice thickness of 400 μm. The slices were then incubated in oxygenated (95% O_2_/5% CO_2_) artificial cerebrospinal fluid containing (in mM) 240 sucrose, 2.5 potassium chloride, 1.25 sodium dihydrogen phosphate, 25 sodium bicarbonate, 0.5 calcium chloride, 3.5 magnesium chloride, 0.4 sodium ascorbate, and 2 sodium pyruvate, pH 7.35, 310 mOsm for at least 1 h. The slices were subsequently transferred to a chamber and continuously perfused with oxygenated (95% O_2_/5% CO_2_) artificial cerebrospinal fluid at a rate of 0.5–1 mL/min, with the perfusion solution containing (in mM): 117 sodium chloride, 3.6 potassium chloride, 1.2 sodium dihydrogen phosphate, 11 glucose, 25 sodium bicarbonate, 2.5 calcium chloride, 1.2 magnesium chloride, 0.4 sodium ascorbate, and 2 sodium pyruvate, pH 7.35, 310 mOsm. Glass microelectrodes were pulled using an MP‐500 microelectrode puller (RWD Life Science, Shenzhen, China) and filled with an intracellular solution containing the following (in mM): 135 cesium chloride, 15 sodium chloride, 5 tetraethylammonium chloride, 2 magnesium chloride, 10 HEPES, 0.6 EGTA, and 5 ATP, pH 7.35, 290 mOsm. Whole‐cell recordings with a seal resistance greater than 3 GΩ were obtained from CA1 pyramidal cells. The artificial cerebrospinal fluid was supplemented with 0.5 mM TTX, 10 mM CNQX, 40 mM APV, and 100 nM L‐655,708 (MedchemExpress, NJ, USA) dissolved in the artificial cerebrospinal fluid for testing. Miniature inhibitory postsynaptic currents (mIPSCs) were recorded using Clampex 10 (Molecular Devices, San Jose, CA) at a 2 kHz filtering frequency and stored on videotape for offline analysis using Clampfit 10 (Molecular Devices, San Jose, CA). The amplifier used was an Axon MultiClamp 700B (Molecular Devices), and the analog‐to‐digital converter used was a Digidata 1440A (Molecular Devices).

### 2.11 Statistical Analysis

All the results are presented as the means ± SEMs. Unpaired Student's *t*‐tests were used to compare two groups. For comparisons involving four groups, one‐way analysis of variance (ANOVA) and Bonferroni's post hoc correction were employed. The experimental data were normally distributed. Statistical analyses were conducted using GraphPad Prism 8.0, with *p* < 0.05 indicating statistical significance in this study.

## 3 Results

### 3.1 Fasudil Ameliorates Cognitive Function Impairment in Mice Induced by Anesthesia and Surgery

We first established the mouse model of PND and analyzed arterial blood gases after surgery to exclude the possibility of respiratory depression caused by anesthesia. No statistically significant differences were observed among the groups of mice (Table 2), indicating that the mice were safe during the surgical procedure.

**TABLE 2:** Postoperative analysis of arterial blood gas parameters in mice.

Arterial blood gas analysis in mice (*n* = 4)					
Group	NS + CON	FSD + CON	NS + SUR	FSD + SUR	ANOVA *p*
pH	7.329 ± 0.03	7.367 ± 0.13	7.389 ± 0.08	7.365 ± 0.04	0.7719
PaCO_2_ (mmHg)	41.1 ± 4.12	39.55 ± 4.95	35.95 ± 6.02	39.53 ± 5.10	0.7672
PaO_2_ (mmHg)	106.8 ± 1.71	109.5 ± 7.33	115 ± 6.83	108.3 ± 3.5	0.2022
SaO_2_ (%)	95.45 ± 2.91	95.28 ± 4.86	98.5 ± 1.25	96.05 ± 0.94	0.4015

We then used behavioral tests to evaluate the cognitive function of each group of mice and assess whether our fracture model caused a postoperative cognitive impairment in mice. During the 5‐day training phase in the water maze, the escape latency of the mice gradually decreased, indicating a deeper memory of the platform location, with no significant differences in learning ability observed among the groups (Figure 1E). In the testing phase, the time spent by the mice that underwent fracture surgery in the quadrant where the platform was located was significantly shorter than that spent by mice in the control group (Figure 1G,H). Additionally, the escape latency, which represents the time taken to reach the original platform location, was significantly longer in the fracture surgery group than in the control group (Figure 1F), whereas the swimming speed was not significantly different (Figure 1J). The results of the contextual fear condition test indicated no statistically significant differences in freezing time among the groups (Figure 1M), but a significant difference was observed in contextual freezing time between the fracture surgery group and the control group (Figure 1L). In this study, the freezing time of the mice in the fracture group was shorter than that of the control group. The open‐field test results revealed no differences in the average movement speed of the mice among the groups (Figure 1K), indicating that the postoperative motor abilities of the mice were restored.

During the MWM testing phase, mice in the fracture surgery group that were pretreated with fasudil exhibited a recovery of the time spent in the quadrant where the platform was located and the number of crossings, and the escape latency returned to baseline values (Figure 1F–H). In the FCT, no statistically significant differences in freezing time in response to the tone were observed between the groups, but mice in the fracture surgery group that were pretreated with fasudil presented an increase in the contextual freezing time compared with those in the simple fracture group (Figure 1L,M). These findings indicate that pretreatment with fasudil improved postoperative cognitive function in mice.

### 3.2 Fasudil Restores the Increase in α5GABAAR Expression and Function Caused by Anesthetic Surgery

**FIGURE 2:**
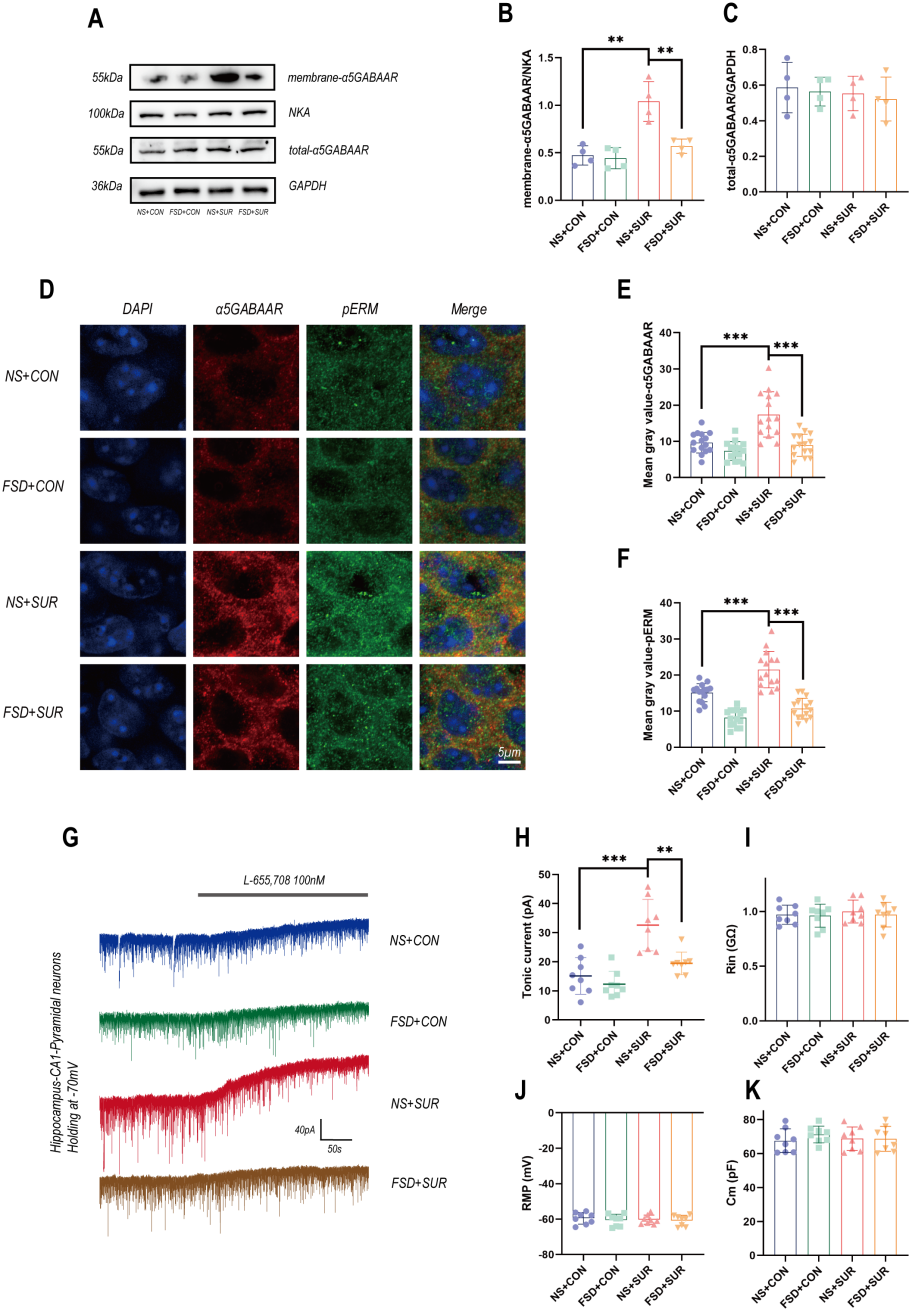
Changes in the expression and function of α5GABAAR. (A–C) Quantification of the cell membrane and whole‐cell α5GABAARs (*n* = 5) levels using Western blotting. (D) Immunofluorescence staining for α5GABAAR (red) and pRadixin (green). (H–J) Bar graphs displaying intergroup variations (*n* = 5, with three brain slices taken from each mouse). (G–K) Patch‐clamp recordings were performed to assess the function of α5GABAAR in the pyramidal neurons of the hippocampal CA1 region in acute brain slices. (G) Following the perfusion of L‐655,708, blockade of α5GABAAR in neurons resulted in an increase in the tonic current, which was attributed to the blockade of extrasynaptic α5GABAAR. (H) Histogram illustrating the changes in the tonic current in each group (*n* = 4, with two brain slices taken from each mouse). (I) After sealing, the membrane resistance of each cell was recorded to eliminate the influence of differences in cell size on the current. (J) The resting membrane potential of each cell after sealing. (K) The membrane capacitance of each cell after sealing. The data are presented as the means ± SEMs, *p < 0.05, **p < 0.01, and ***p < 0.001 (unpaired Student's *t*‐test).

Previous studies have shown that the upregulation of α5GABAARs on the cell membrane in the hippocampus promotes PND [10, 19, 20, 21, 22, 23, 24]. To evaluate the changes in the quantity of α5GABAARs, we first used Western blotting to detect the membrane proteins extracted from the hippocampal region. The electrophoresis results revealed a significant upregulation of α5GABAAR expression on the membrane in the fracture surgery group, which was effectively inhibited by fasudil treatment, whereas a statistically significant difference in α5GABAAR expression in the total hippocampal protein was not observed after surgery (Figure 2A–C). We subsequently conducted immunofluorescence staining on mouse brain slices. We did not use Triton X‐100 for permeabilization to detect the expression of receptors on the membrane. The immunofluorescence results revealed that α5GABAAR was expressed mainly on the cell membrane of somatic neurons in the hippocampus. After fracture surgery, the expression of α5GABAAR around the cell bodies of neurons in the CA1 region of the hippocampus increased. In mice pretreated with fasudil, the expression of α5GABAAR returned to baseline levels (Figure 2D,E). Tonic currents were detected to reflect the function of α5GABAAR. The electrophysiological results revealed a significant increase in tonic currents in the mice in the surgery group compared with those in the control group, while tonic currents in the fasudil pre‐treatment group were restored to physiological levels (Figure 2G,H). These experimental results suggest that fasudil improves the neural function of PND mice by decreasing the abnormal increase in the membrane expression of α5GABAARs induced by anesthesia and surgery.

### 3.3 The Expression of Surface α5GABAARs is Simultaneously Regulated by Both the Camk2 and Radixin Signaling Pathways

**FIGURE 3:**
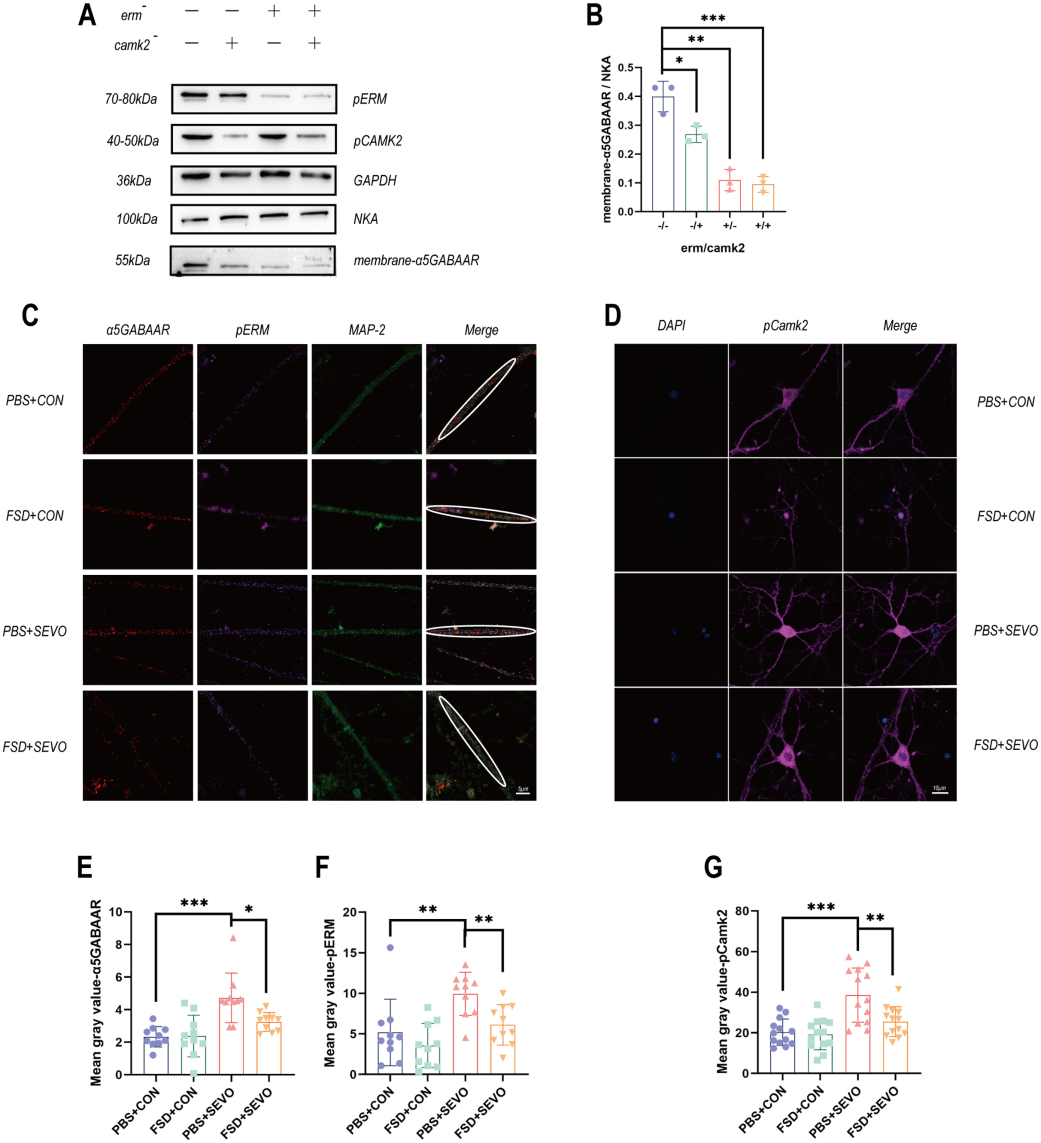
The impact of two pathways on α5GABAAR at the cellular level. (A, B) Changes in protein levels in HT‐22 cells (*n* = 3). (B, C) Semiquantitative immunofluorescence analysis of primary mouse hippocampal neurons. (C) Expression and distribution of α5GABAAR (red) and pRadixin (purple) on neuronal dendrites. (D) Overall expression of pCamk2 (purple) in neurons. (E, F) Bar graphs showing the results of the semiquantitative analysis of α5GABAAR and pRadixin expression on dendrites among the different groups (*n* = 10). (G) Semiquantitative analysis of overall pCamk2 expression in neurons (*n* = 13). The data are presented as the means ± SEMs, *p < 0.05, **p < 0.01, and ***p < 0.001 (unpaired Student's *t*‐test).

The expression of α5GABAARs was reported to be regulated by radixin or Camk2 pathway. We further employed siRNAs to silence Radixin and Camk2 expression separately in the HT‐22 cell line to verify whether a single pathway affects the aggregation of α5GABAARs on the membrane and to determine the regulatory role of both the Ca^2+^/Camk2 and ROCK II/Radixin signaling pathways in the expression of α5GABAARs on the cell membrane. The Western blot results revealed that silencing the expression of Radixin and Camk2 individually had inhibitory effects on the aggregation of α5GABAARs on the cell membrane (Figure 3A,B).

### 3.4 Fasudil Suppressed the Over‐Expression of α5GABAARs via Modulation of Radixin and Camk2 Pathways

To further investigate the effects of sevoflurane and fasudil on the expression of membrane α5GABAARs via the aforementioned pathways, we extracted primary hippocampal neurons from mice for validation. First, we subjected primary hippocampal neurons to sevoflurane anesthesia. We conducted immunofluorescence staining on DIV14 neurons to examine whether sevoflurane increased the surface expression of α5GABAARs in neurons. The results revealed a statistically significant increase in the expression of α5GABAARs on the dendrites of neurons after exposure to anesthesia alone, with increased phosphorylation of ERM observed following anesthesia exposure (Figure 3C,E,F). Overall, a significant increase in Camk2 phosphorylation was observed in neurons after anesthesia exposure (Figure 3D,G). These findings indicate that at the cellular level, exposure to anesthesia alone can activate both pathways, leading to the aggregation of α5GABAARs on the membrane. Administration of the dual‐pathway inhibitor fasudil 24 h before anesthesia exposure resulted in the inhibition of ROCK II/Radixin and Ca^2+^/Camk2, returning α5GABAARs expression to baseline levels (Figure 3C,E,F).

### 3.5 Fasudil Inhibited the Activation of Radixin and Ca^2+^/Camk2 Dual Pathways in PND Mouse Models

**FIGURE 4:**
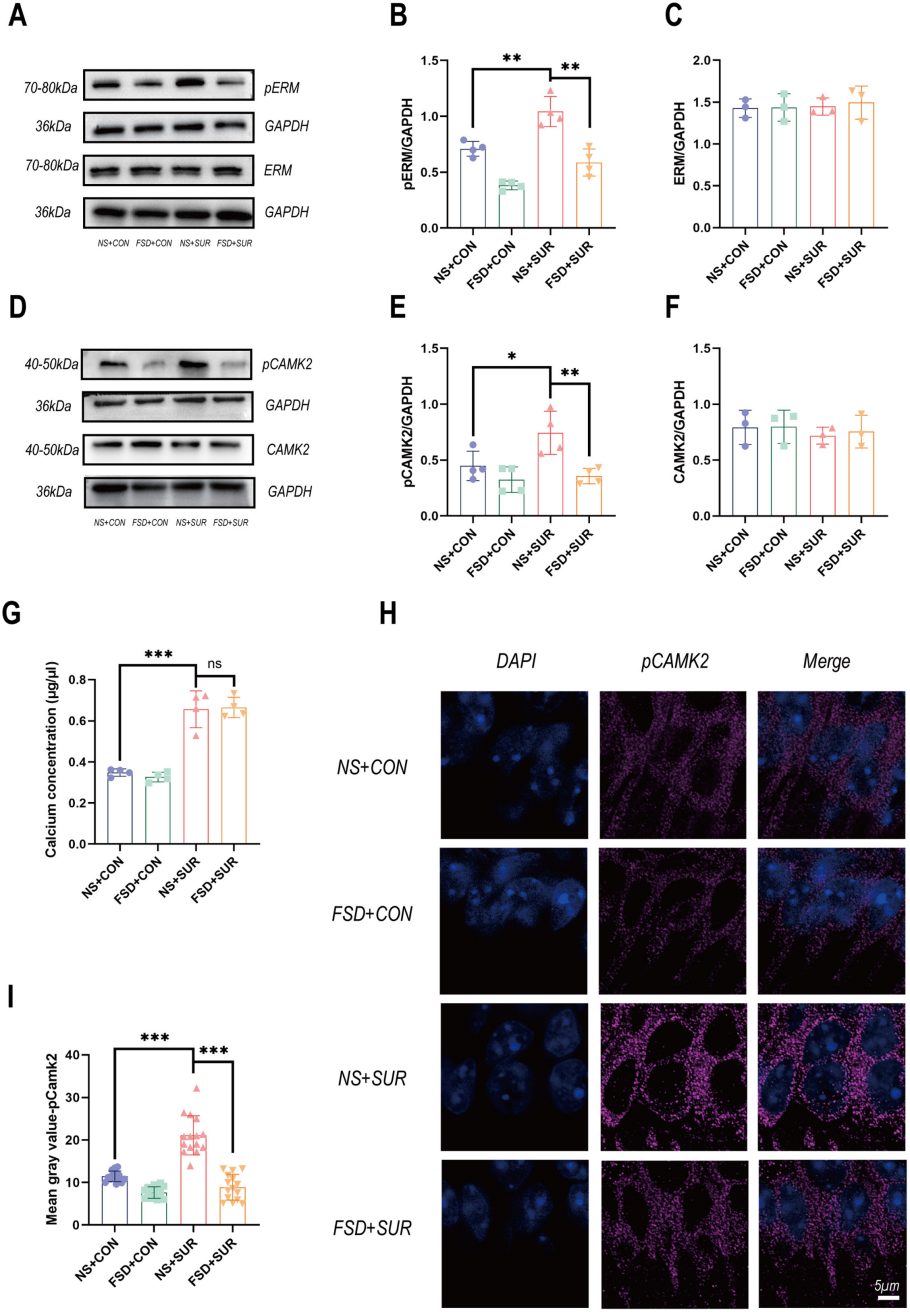
Changes in p‐ERM and p‐Camk2 levels after surgery. (A–C) Quantification of the changes in levels of the anchoring protein Radixin (*n* = 3) and its phosphorylation (*n* = 4) in mouse hippocampal tissue using Western blotting. (D–F) Quantification of the level of the transfer protein Camk2 (*n* = 3) and its phosphorylation (*n* = 4) using Western blotting. (ERM, CAMK2 and GAPDH* in “Figure 4A,D” are from the same gel) (G) Quantitative analysis of the calcium concentration in mouse hippocampal tissue (*n* = 4). (H) Immunofluorescence staining pCamk2 (purple) in the pyramidal neurons of the hippocampal CA1 region in acute brain slices. (I) Bar graphs displaying intergroup variations (*n* = 5, with three brain slices taken from each mouse). The data are presented as the means ± SEMs, *p < 0.05, **p < 0.01, and ***p < 0.001 (unpaired Student's *t*‐test).

Our previous research demonstrated that sevoflurane increases the expression of α5GABAARs on the membrane in aged mice through ROCK II/Radixin [28]. We examined ROCK II/Radixin protein expression in 8‐week‐old mice to demonstrate the activation of ROCK II/Radixin in young mice. We found that after anesthesia and surgery, the levels of ROCK II and p‐Radixin in the mice were significantly elevated, whereas the total amount of Radixin was not significantly different (Figure 4A–C). The immunofluorescence results revealed a significant increase in p‐Radixin expression around the cell bodies of neurons in the CA1 region of the hippocampus after fracture surgery (Figure 4D,F). Considering that some studies have indicated that inhalational anesthetics cause the opening of the ryanodine receptor, leading to an increase in intracellular Ca^2+^ concentrations [29]. We conducted tests for calcium ions and the Camk2 protein in the hippocampus of the mice. Compared with that in the control group, the concentration of calcium ions in the hippocampus of the mice after fracture surgery was significantly increased (Figure 4G), and the phosphorylation of Camk2 in the hippocampal protein of the mice was increased after surgery, whereas the overall expression of Camk2 was not significantly different (Figure 4D–F). Immunofluorescence results revealed an increase in Camk2 phosphorylation in pyramidal neurons in the CA1 region after fracture surgery (Figure 4H,I). These results indicate that anesthesia and surgery activate the ROCK II/Radixin and Ca^2+^/Camk2 pathways in the CA1 region of the hippocampus in mice.

Western blot and immunofluorescence results indicate that fasudil inhibits the activation of the ROCK II/Radixin and Ca^2+^/Camk2 dual pathways induced by anesthesia and surgery (Figures 2D–F, 4H,I). By suppressing the phosphorylation of Radixin and Camk2, fasudil significantly reduces the increase in surface expression of α5GABAARs on the cell membrane caused by anesthesia and surgery. These findings suggest that fasudil, through its inhibitory effects on Camk2 and ROCK, attenuates the phosphorylation of Camk2 and Radixin, thereby diminishing the abnormal expression of α5GABAARs on the membrane in PND mice. Our preliminary results indicate that Fasudil inhibits the activation of Radixin and Ca^2+^/Camk2 dual pathways in PND mouse models. Through this mechanism, Fasudil may have a preventive effect on PND in mice.

## 4 Discussion

**FIGURE 5:**
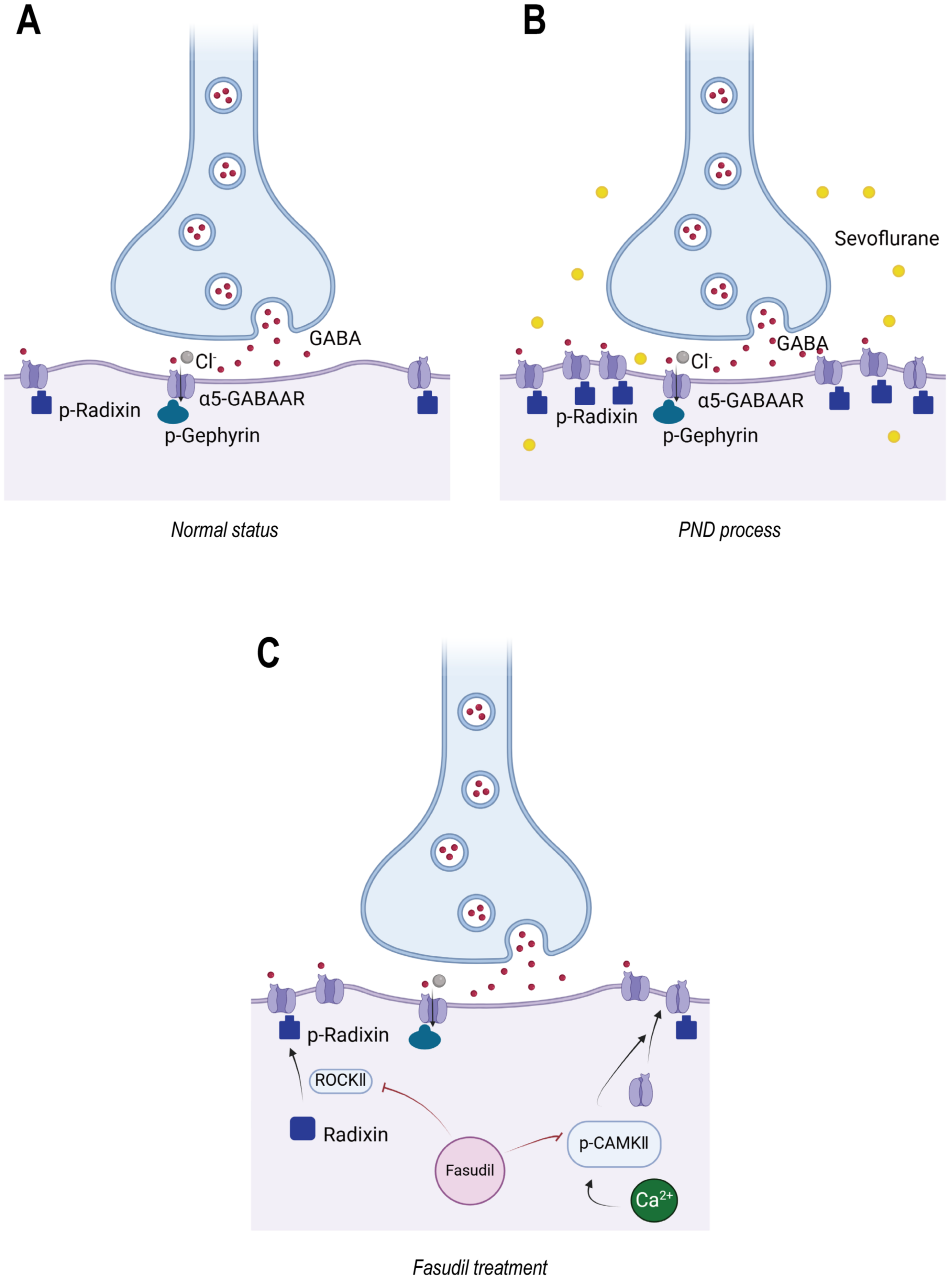
(A) The Function of α5GABAARs in Normal State. (B) Anesthesia and surgery increase the calcium concentration in the hippocampus, increasing the phosphorylation of Camk2. Anesthesia and surgery activate the Radixin pathway, leading to increased phosphorylation of Radixin. The activation of these two pathways results in increased surface expression of α5GABAARs, ultimately leading to a hippocampus‐dependent memory impairment. (C) Preadministration of the neuroprotective drug fasudil can inhibit the phosphorylation of the key molecules mentioned above, prevent the translocation of α5GABAARs to the cell surface, and thereby mitigate neurocognitive damage.

In recent years, the use of anesthetics, one of the most significant advancements in modern surgical practice, holds far greater therapeutic value for surgical assistance and patient care than the side effects it may induce. With the development of comfort medicine, early postoperative recovery is the goal of all physicians. Consequently, PND, one of the most common complications after anesthesia, is a pressing issue in need of resolution. In the current study, we found that following a 2‐h exposure to 4.3% sevoflurane anesthesia and subsequent femoral fracture internal fixation surgery in mice, increased phosphorylation of Radixin and Camk2 was observed in the hippocampal CA1 region, along with increased expression and function of α5GABAARs in the extrasynaptic cell membrane, leading to impaired cognitive function in mice. However, preadministration of fasudil attenuated the phosphorylation of Radixin and Camk2, thereby reducing the expression of α5GABAARs on the cell membrane and significantly protecting cognitive function (Figure 5).

Due to advancements in patch‐clamp techniques, the aberrant function of α5GABAARs has been extensively linked to alterations in learning and memory [10, 19, 20, 21, 22, 23, 24, 39, 40]. Previous studies have demonstrated that an abnormal elevation in the functionality of α5GABAARs is associated with the inhalation of volatile anesthetics [41]. Orser's extensive work [10, 19, 20, 21, 22, 23, 24] confirmed that an elevation in α5GABAAR function is one of the main causes of PND. In a study from 2012 [24], Orser and colleagues reported that sevoflurane failed to induce long‐term memory impairments in animal models lacking α5GABAAR gene mutations. And numerous α5GABAAR inhibitors, such as L‐655,708, α5IA, and S44819 [24, 42, 43], have been utilized to improve cognitive function. These findings further underscore the pivotal role of αGABAAR in cognitive function. In our current study, we observed that cognitive impairment occurred effectively in young mice 72 h after anesthesia and surgery. In mice with PND induced by anesthesia and surgery, an increase in α5GABAAR expression was detected in the hippocampal region, with enhanced function of the α5GABAAR in mediating chloride ion influx extracellularly. The increased expression and function of the receptor after surgery align with the findings reported by Orser and colleagues.

In our previous work [28], we elucidated the role of the ROCK II/Radixin pathway in the process of the sevoflurane‐induced increase in α5GABAAR function in the hippocampal region of aged mice, which is also reflected in the results of this study. Hausrat et al. [27] reported that increasing pRadixin expression facilitates the clustering of extrasynaptic α5GABAARs, whereas the behavioral results revealed impaired learning and memory functions in mice, supporting our data. The ROCK/Radixin pathway was activated in the hippocampus of mice with PND induced by anesthesia and surgery in this study, leading to increased phosphorylation of the anchoring protein Radixin. In addition, the concentration of calcium ions can also be affected by anesthetics. Liu et al. [29] proposed that the increase in calcium ion concentrations caused by anesthesia may be related to the opening of ryanodine receptors induced by anesthetics. Camk2, a calcium‐regulated protein, can facilitate the movement of GABARs to the cell membrane. Saliba, Kretschmannova, and Moss confirmed [32] that the activation of Camk2 plays a role in the aggregation of α5GABAARs on the membrane, which provided guidance for this study. We examined the calcium ion levels in the hippocampus of postoperative mice and revealed that the calcium concentration in the postoperative hippocampus was higher than that in the control mice. Additionally, an increase in the phosphorylation of Camk2 was detected. The results indicate that the increase in calcium ion concentrations after anesthesia can promote the translocation of α5GABAAR from the cytoplasm to the cell membrane through the phosphorylation of Camk2.

This study revealed that anesthesia and surgery activate both the ROCK II/Radixin and Ca^2+^/Camk2 pathways in the mouse hippocampus, leading to increased expression and function of synaptic α5GABAARs in CA1 pyramidal neurons. This process is a key factor in the occurrence of PND. As an inhibitor targeting the aforementioned pathways, fasudil has already been clinically employed, ensuring the safety of this medication [34, 35]. Fasudil, known as a brain protectant, is indicated for cerebral vasospasm and ischemic symptoms following subarachnoid hemorrhage. Fasudil has been extensively studied and shown to have positive effects on various central nervous system disorders [35, 36, 37, 44, 45, 46, 47]. However, no reported evidence is available on its preventive or therapeutic effects on PND. In this study, we demonstrate that prophylactic administration of fasudil can improve postoperative cognitive function in mice by inhibiting ROCK, thereby reducing the anchoring of α5GABAARs on the postsynaptic membrane. Additionally, through its inhibition of Camk2, fasudil reduces the clustering of α5GABAARs on the membrane, providing a secondary mechanism for protecting postoperative cognitive function in mice. However, fasudil did not decrease the calcium concentration in the hippocampus. We hypothesize that fasudil acts by directly inhibiting the phosphorylation of CaMKII, thus not impacting calcium ion concentration.

The balance between excitatory and inhibitory receptors at synapses affects the overall function of brain regions. One limitation of this study is that only inhibitory receptors were analyzed. Additionally, crosstalk between neurons and both astrocytes and microglia may influence the transmission and modulation of GABA signaling [48], which could be further elucidated in future exploratory studies to elucidate the delicate balance among anesthetics, GABA, and GABARs.

## 5 Conclusion

In conclusion, our preliminary results suggest that preadministration of fasudil improved postoperative cognitive function in PND mice by inhibiting the activation of the Camk2 and Radixin pathways and finally downregulating the surface expression of α5GABAAR in hippocampus neurons. As an already clinically employed medication, fasudil may offer more options for the prevention and treatment of postoperative neurocognitive dysfunction in the future.

## Author Contributions

Y.Y., K.W., and Y.W. designed the experiments. J.D. and Z.W. contributed to writing the manuscript. J.D., Z.W., L.L., and M.Z. contributed to performing the experiments. Y.D. and S.W. contributed to the data analysis. X.W. and Y.L. contributed to editing the images and revising the manuscript. All authors contributed to the article and approved the submitted version.

## Conflicts of Interest

The authors declare no conflicts of interest.

## Data Availability

The data that support the findings of this study are available from the corresponding author upon reasonable request.
